# The Influence of miRNAs on Radiotherapy Treatment in Prostate Cancer – A Systematic Review

**DOI:** 10.3389/fonc.2021.704664

**Published:** 2021-08-03

**Authors:** Sílvia Soares, Susana G. Guerreiro, Natália Cruz-Martins, Isabel Faria, Pilar Baylina, Maria Goreti Sales, Miguel A. Correa-Duarte, Rúben Fernandes

**Affiliations:** ^1^ BioMark@ISEP, School of Engineering, Polytechnic Institute of Porto, Porto, Portugal; ^2^ LaBMI – Laboratory of Medical & Industrial Biotechnology, Porto Research, Technology & Innovation Center (PORTIC), P.PORTO – Polytechnic Institute of Porto, Porto, Portugal; ^3^ Institute for Research and Innovation in Health (i3S), Porto, Portugal; ^4^ Faculty of Chemistry, University of Vigo, Vigo, Spain; ^5^ CEB, Centre of Biological Engineering of Minho University, Braga, Portugal; ^6^ Institute of Biomedical Sciences Abel Salazar, University of Porto, Porto, Portugal; ^7^ Institute of Molecular Pathology and Immunology of the University of Porto-IPATIMUP, Porto, Portugal; ^8^ Department of Biomedicine, Biochemistry Unit, Faculty of Medicine, University of Porto, Porto, Portugal; ^9^ Institute of Research and Advanced Training in Health Sciences and Technologies (CESPU), Gandra, Portugal; ^10^ School of Health, Polytechnic of Porto, Porto, Portugal; ^11^ Biomark@UC, Department of Chemical Engineering, Faculty of Sciences and Technology, University of Coimbra, Coimbra, Portugal; ^12^ CINBIO, University of Vigo, Vigo, Spain; ^13^ Southern Galicia Institute of Health Research (IISGS), and Biomedical Research Networking Center for Mental Health (CIBERSAM), Vigo, Spain

**Keywords:** prostate cancer, radiotherapy, RNA therapy, microRNA, oncomiR, oncosupressor miR

## Abstract

In the last years, extensive investigation on miRNomics have shown to have great advantages in cancer personalized medicine regarding diagnosis, treatment and even clinical outcomes. Prostate cancer (PCa) is the second most common male cancer and about 50% of all PCa patients received radiotherapy (RT), despite some of them develop radioresistance. Here, we aim to provide an overview on the mechanisms of miRNA biogenesis and to discuss the functional impact of miRNAs on PCa under radiation response. As main findings, 23 miRNAs were already identified as being involved in genetic regulation of PCa cell response to RT. The mechanisms of radioresistance are still poorly understood, despite it has been suggested that miRNAs play an important role in cell signaling pathways. Identification of miRNAs panel can be thus considered an upcoming and potentially useful strategy in PCa diagnosis, given that radioresistance biomarkers, in both prognosis and therapy still remains a challenge.

## Introduction

Small non-protein-coding RNA molecules, composed of around 22 nucleotides, are commonly named as miRNAs (1–3). Briefly, miRNAs are expected to account for 1-5% of the human genome and to interfere with at least 30% of the protein-coding genes (4, 5). The first miRNA was discovered in 1993 by Lee, Freinbaum and Ambros (6, 7), and since then an increasing load of literature data have pointed that they can act as both tumor suppressors and oncogenes (1–3). Indeed, it has been shown that miRNAs play an important role in gene expression, mainly when associated with the monitoring of several cell and metabolic pathways, being also an essential component of the gene silencing machinery in most eukaryotic organisms (4, 8).

In recent years, many studies have confirmed the involvement of miRNAs in biological processes of several types of cancer (4, 9, 10). The relationship between miRNAs and cancer was demonstrated for the first time in 2002, with miRNAs being stated as a potential mechanism that may contribute to improve some cancer therapeutic approaches through restoring or blocking the miRNAs function (11). Among the various types of cancer with increasing prevalence nowadays, prostate cancer (PCa) is the second most common in male and the fifth leading cause of death in men. Based on Global Cancer Observatory (GLOBOCAN) 2020, more than 1.4 million new cases of PCa and 375,304 associated deaths were recorded (12). One of the treatments applied in cancer is radiotherapy (RT), a therapeutic modality that uses ionizing radiation to induce damage in unwanted cells. The main goal of RT consists in delivering a precise dose of radiation in a target volume, such as tumor, promoting the tumor cells eradication with as minimal damage as possible in surrounding normal tissues (13). Currently, RT is one of the most often used therapeutic approaches in PCa patients, featured by several levels of complexity (13, 14), with around 50% of all PCa patients receiving RT at some stage of treatment, while 10–45% of PCa cases are resistant to irradiation (15, 16). Besides the RT dose is standardized among patients, local recurrences are common and can occur even when modern techniques are used (17). Patient’s local recurrences appear mostly as a result of uncontrolled cell reproduction and unregulated cancer cells growth that invades and interferes with the normal function of surrounding tissues and organs (1). Various regulatory factors and genes have shown to be able to directly modulate cell cycle, differentiation and even death. For instance, tumor-suppressor genes or oncogenes or both are regulatory factors able to modulate the environmental conditions contributing to cancer development (2, 4, 18). In this way, miRNAs may be viewed as promising biomarkers capable of predicting radiation response and to develop a customized treatment for each patient, ultimately opening a new therapeutic window for personalized intervention in PCa patients.

Therefore, in the present work, we aimed to explore the mechanisms of miRNA biogenesis, the role of miRNAs in cancer, and the functional impact of miRNAs on PCa radiation response, towards to provide a detailed review of the miRNA expression signatures in PCa tumor and cell lines with therapeutic impact in RT.

## Methods

**Figure 1:**
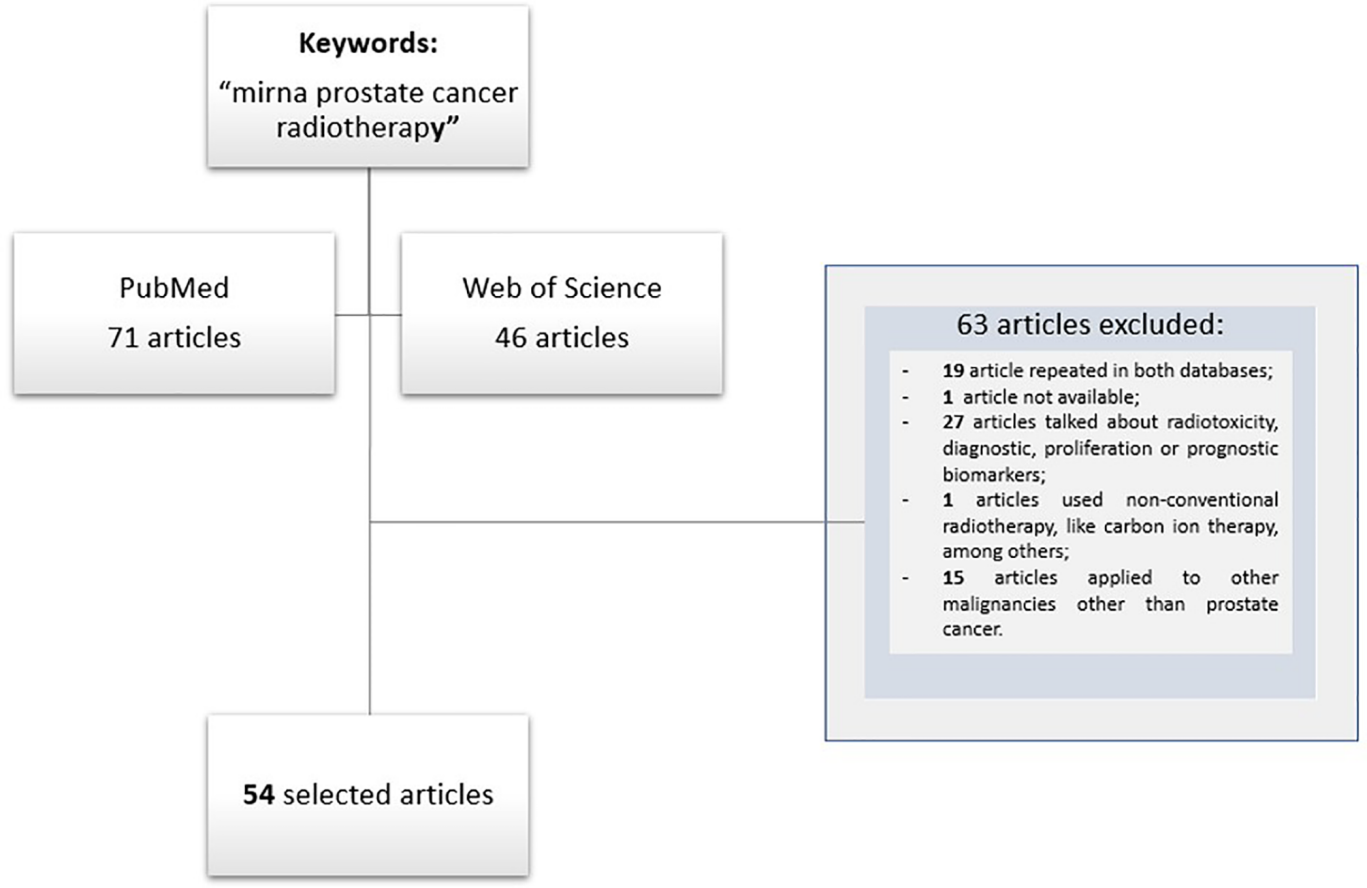
Flow diagram of studies selection.

For data selection ‘PICOS Worksheet and Search Strategy’ was followed. A detailed and careful literature search was done using PubMED and Web of Science databases. For the Boolean search combinations, the terms used were “miRNA AND prostate cancer OR prostate neoplasia OR prostate carcinoma AND radiotherapy OR radiation therapy”, resulting in 71 papers from PubMED and 46 from “Web of Science”. Inclusion criteria include articles written in English, published between January 2000 and June 2021, and works referred to clinical studies and pre-clinical studies with cell lines and animal models. Exclusion criteria include not repeat articles on different databases, articles not available and papers that employ non-conventional RT, that with focus on radiotoxicity, and papers on radio sensitization-related biomarkers applied for either diagnostic or prognostic purposes. Then, these 117 articles were analyzed by independent researchers, and 54 articles were selected for further analysis, with 63 articles being eliminated because did not fulfil with the inclusion criteria. Papers related to other malignancies in addition to PCa and those in whom was unable to obtain the whole data were also excluded (**Figure 1**).

## Results and discussion

### miRNA Biogenesis

**Figure 2:**
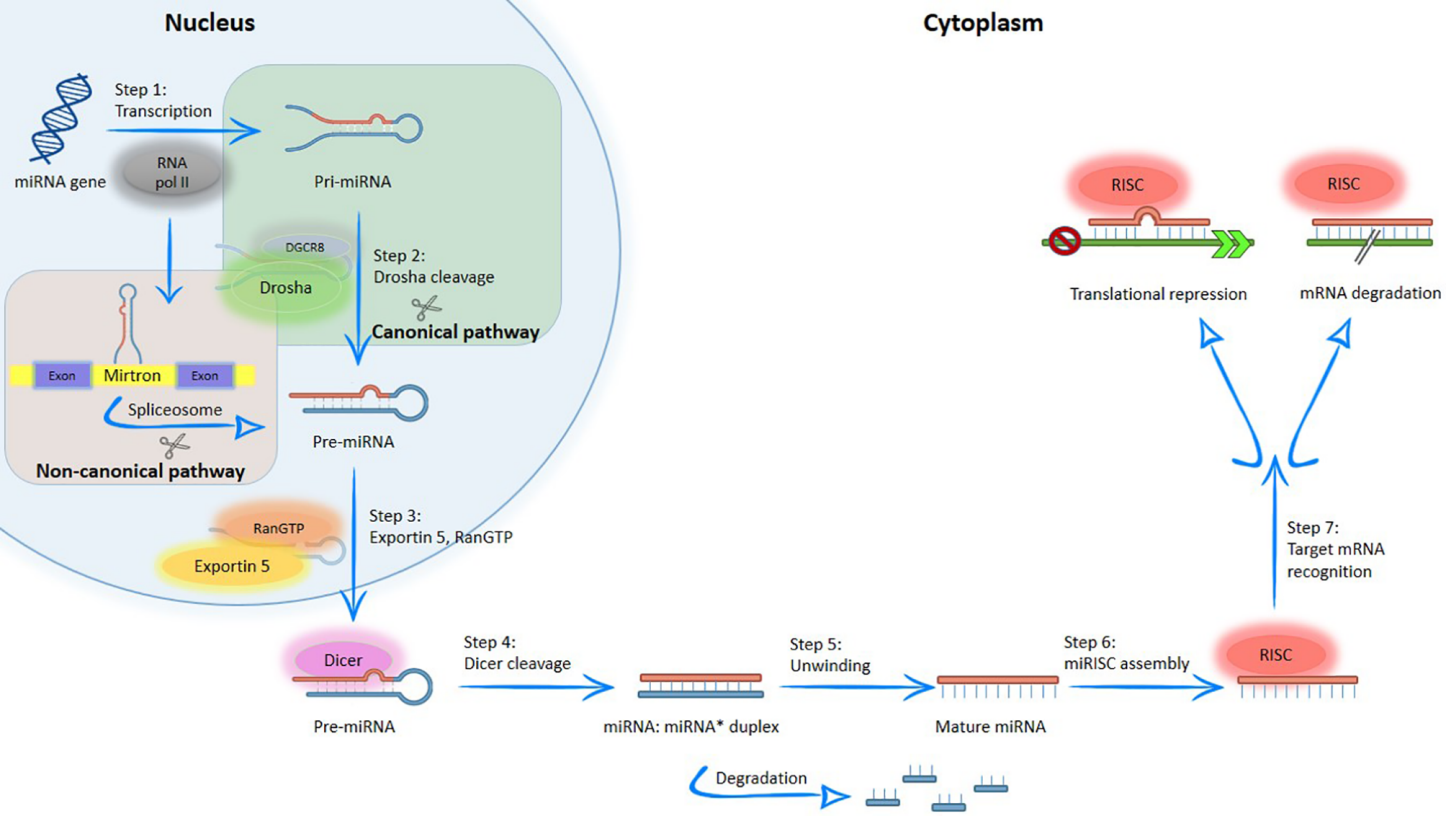
Simplified overview of the canonical and non-canonical miRNA biogenesis pathways. MiRNA generated by canonical pathway is transcribed by RNA polymerase II to a primary transcript called pre-miRNA. Subsequently, this structure is processed into the nucleus by DGCR8/Drosha complex; producing the pre-miRNA, which is exported to the cytoplasm by exportin-5. In the cytoplasm, pre-miRNA is cleaved by RNase III endonuclease (Dicer protein), resulting in a double stranded miRNA, which contains the mature miRNA. After, double strand is separated by helicases and create the mature miRNA; on the contrary, in non-canonical pathways, miRNA located within of mirtrons and are associated with spliceosome-dependent mechanisms. After, the mature miRNA produced by different pathways enters into the RISC complex and regulated genes through messenger RNA (mRNA) degradation and mRNA translation repression.

miRNA biogenesis is controlled at multiple steps, and transcriptional regulation has been proposed to be the major mechanism controlling tissue and cell type-specific expression of miRNAs (4, 19). Briefly, the miRNAs biogenesis includes their transcription at cell nucleus, export to the cytoplasm and subsequent processing and maturation (20), with two processes being involved in achieving the mature miRNA: canonical or non-canonical biogenesis of miRNA (**Figure 2**).

In the canonical pathway, the miRNA gene is typically transcribed by RNA polymerase II to generate long primary transcripts (pre-miRNA) in the nucleus. Subsequently, the pre-miRNA is processed by RNA polymerase III (Drosha protein) and DiGeorge syndrome critical region gene 8 (DGCR8) protein, thus producing the pre-miRNA, a double-stranded miRNA of variable length with approximately 18-25 nucleotides (4, 5, 19, 21, 22). The resulting structure is exported to the cytoplasm *via* Exportin-5 and RanGTP (5, 19, 21, 23). In the cytoplasm, pre-miRNA is cleaved by the Dicer protein to create a duplex miRNA, which contains the mature miRNA. When the duplex unwinds, both RNA strands are separated by helicases and the resulting mature miRNA is incorporated into a functional ribonucleoprotein complex, called RNA-Induced Silencing Complex (RISC), while the other strand is degraded (4, 9, 19). RISC is a ribonucleoprotein complex composed by a set of proteins linked to a small molecule of RNA and it is responsible to perform cell surveillance, inhibiting the translation of the gene into a protein through enzymatic destruction, which effectively silences the gene (5). Both miRNA and RISC complex (miRISC) regulate gene expression through two mechanisms: messenger RNA (mRNA) degradation and mRNA translation repression (22, 23).

Non-canonical pathway is an alternative biogenesis pathway, where miRNA is associated with spliceosome-dependent mechanisms (19). In this pathway, the miRNAs located within the introns of coding or non-coding genes of proteins (“mirtrons”) enter in the miRNA processing pathway without Drosha-mediated cleavage (23).

### miRNAs in Cancer Hallmarks

**Figure 3:**
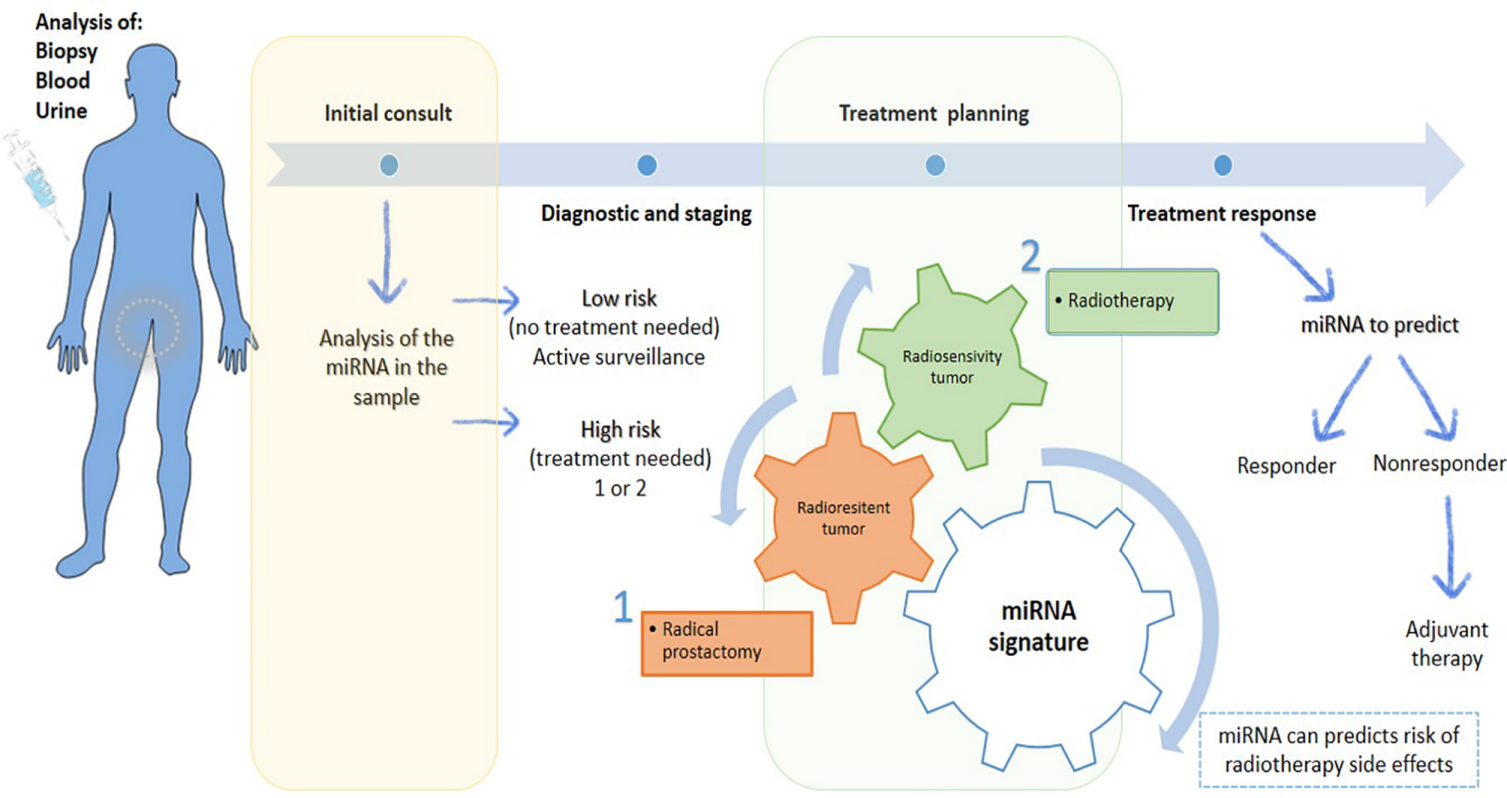
Scheme of the potential of miRNAs in personalized prostate cancer. MiRNAs are present in biological samples, which could be a useful tool for diagnosis and staging in the first consult, allowing an accurate risk stratification. Based on information collected, the treatment can be planned. Treatments can be personalized according to radioresistance or radiosensitive of cancer. If cancer is radioresistance, radical prostatectomy is the therapeutic approach more indicated. Otherwise, RT is ideal treatment to apply to radiosensitive tumors. Also, miRNA signature can give information about the risk of develop side effects or if the patient is responding or not to treatment, leading to a better tumor control with reduced side effects, which contribute to a better patient quality life.

miRNAs can be used in cancer diagnosis to improve the treatment planning and therapeutic sensitivity, to prevent the occurrence of several medications-associated side effects and toxicity, and even to monitor treatment (**Figure 3**). Accumulating evidence underline that miRNAs have an extensive impact due to their involvement in cancer hallmarks, and thus has been considered an important therapeutic target in cancer management (24).

In PCa, there is a dysregulation in miRNAs expression, which can modulate the expression of oncogenes and tumor suppressor genes (20, 22, 23, 25). Moreover, treatment resistance is still a huge problem, so that miRNAs modulation therapy could be a new therapeutic target in cancer patients and used to monitor the therapeutic responses, besides could be useful to predict response to therapies, such chemotherapy and RT (20, 22, 23, 26). The miRNAs therapeutic board approaches include oligonucleotides, small artificial molecules and miRNA-mediated virus or non-virus transfection. In the case of oligonucleotides and small artificial molecules they could be used to inhibit miRNAs or to interfere indirectly with other transcription factors or target genes associated with miRNA-specific modulation. Consequently, several methods have been examined using anti-sense oligonucleotides and it is expected that someday they will be safely implemented (27, 28). Other promissory strategy includes the downregulation of miRNAs using a miRNA-mediated virus or non-virus transfection methods that increases the targeted miRNA. Several studies are being carried out on such matter, taking into account the introduction of artificial double-stranded miRNA – mimic of targeted downregulated miRNA (18).

**Figure 4:**
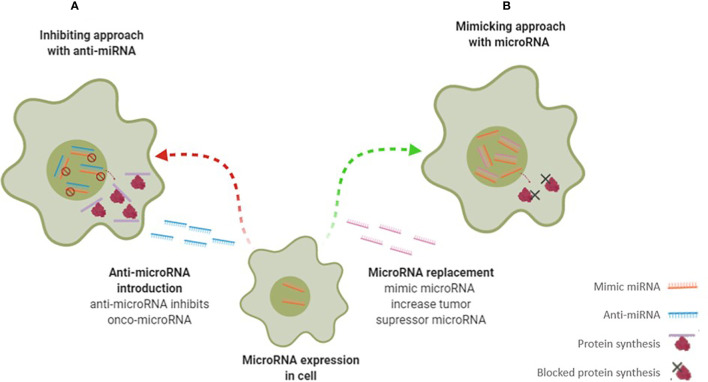
Scheme illustrates different microRNA therapy approaches – anti-microRNA therapy **(A)** and mimicking microRNA therapy **(B)** to change miRNA-regulated gene expression. In therapy **(A)** the microRNA inhibitors are transfected to cells and suppressing onco-microRNA functions and consequently increased protein production. In contrast, in therapy **(B)** are introduced microRNA mimetics to target cells where interact with tumor suppressor microRNA target, suppressing of protein production.

At that time, biological samples, such as blood, serum and urine, allow to classify the cancer risk at same time that provide prognostic data, allowing to address the cancer aggressiveness, predisposition to metastization or development of radio/chemoresistance. Moreover, depending on the cancer risk, an active surveillance or some specific treatments should be recommended. In the last case, miRNAs can help to predict the response to radiation and the likelihood of side effects’ occurrence. So, miRNA expression provides new insights if treatment is being the most appropriate, and if not, treatment must be changed or adjusted (**Figure 4**). Regarding side effects, changes in miRNAs expression can be used to overcome these toxicities or to understand their signs before the need to interrupt the therapy with a possible impairment in therapeutics results (29–31).

### miRNAs as Therapeutic Agents in Radiation Therapy

Currently, the adoption and promotion of personalized therapy has been increasingly notorious, and it is even considered essential in multiple clinical conditions. Specifically, there is increasing evidence underlining those miRNAs can influence the way that cells respond to ionizing radiation, making them more radiosensitive or radioresistant through several specific pathways. These include modifying DNA repair pathways which interfere with cell cycle checkpoints activation, tumor microenvironment and apoptosis. Some miRNAs are involved in controlling cell cycle progression, tumor microenvironment, apoptosis, and radio-related signals pathway (32–34).

In the context of tumor microenvironment, miRNAs have aroused a high interest. MiRNAs play an important role in regulating tumor radiation response, which involve DNA repair, epithelial-to-mesenchymal transition (EMT), and stemness (35–39). However, radio resistance is a complex phenomenon, and thus more studies are needed to better understand such processes. The response rates to radiation differ according to the modality used, namely the way through which radiation is delivered, the dose of radiation used, tumor stage/grade, confounding medical co-morbidities, and intrinsic tumor microenvironment (40, 41).

As mentioned above, miRNAs can be employed in therapeutic approaches to mimic or inhibit gene expression at translation level - **Figure 4** (42, 43). In the first approach, if the miRNA is under expressed, it can be restored by adding miRNA. In the second approach, if the miRNA is overexpressed, artificial anti-miRNAs can be added to block miRNA (29, 33, 42). Preclinical studies have shown that the use of miRNAs is well-tolerated without triggering significant adverse effects. However, it is necessary to improve both the efficiency and targeted delivery to the tumor before treating patients (15).

**Figure 5:**
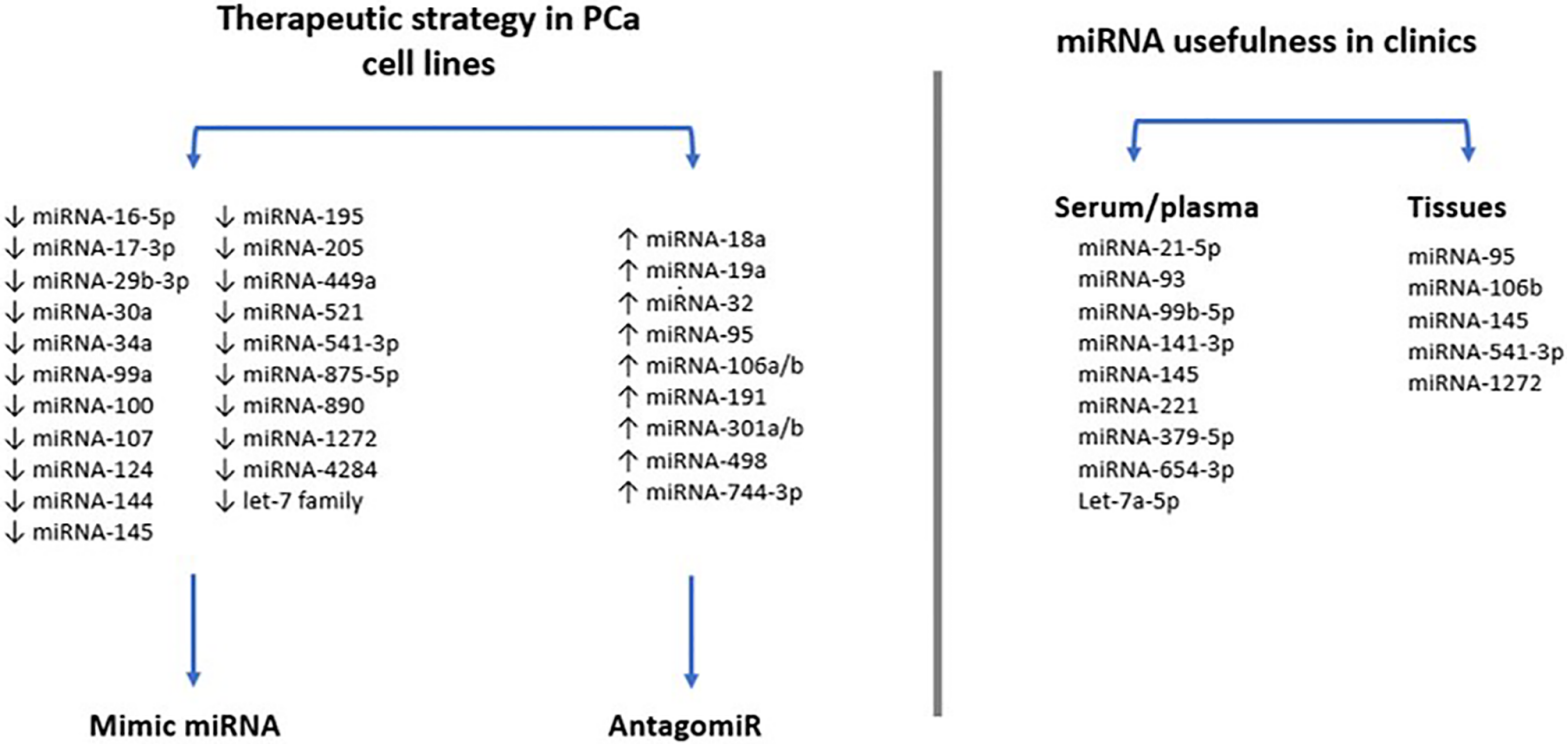
Scheme illustration on the use of miRNAs as a therapeutic strategy in prostate cancer (PCa) cell lines. MiRNAs can be divided into mimic or antagomiR and some have also revealed to be useful in clinics.

To the best of authors’ knowledge, only few clinical trials have investigated the miRNA expression profile induced by RT in PCa patients (**Figure 5**). For example, Zedan et al. measured miRNA-21, miRNA-93, miRNA‐125b, and miRNA‐221 levels in plasma from PCa patients. Among other aspects the authors verified that miRNA-221 and miRNA-93 transcription decreased in patients’ plasma following RT, being thus radiosensitive (44, 45). Also, Linuma et al., in a study where low-dose rate prostate brachytherapy (BT) was applied to PCa patients, they stated that miRNA-93 was significantly downregulated in extracellular vesicles from patients’ serum after BT (34). In addition, miRNA-145 expression was analyzed in tumor tissue of 30 PCa patients and it was suggested that miRNA-145 can improve response to RT reducing the efficiency of the repair of radiation-induced DNA double-strand breaks (DSB). Thus, when miRNA-145 is overexpressed, PCa cells are sensitized to ionizing radiation (46).

Other study verified an upregulation of miRNA-95 in 9 tissue specimens of PCa patients, related to radiation resistance by targeting sphingosine-1-phosphatase 1 (SGPP1) (47). Ambs and colleagues analyzed the miRNA-106b-25 cluster expression in 60 primary PCa and 16 non-tumor PCa tissues and concluded that this miRNA has high levels of expression in primary PCa tissues compared to non-tumor prostate tissues (48). In contrast, miRNA-1272 was found downregulated in PCa tissues (49).

Two other studies investigated miRNAs in extracellular vesicles as markers of therapeutic efficacy. Li et al. found a panel of 9 serum-derived extracellular vesicles-miRNAs (miRNA-200c-3p, miRNA-323-3p, miRNA-379-5p, miRNA-409-3p, miRNA-411-5p, miRNA-493-5p, miRNA-494-3p, miRNA-543, and miRNA-654-3p) with potential to predict the therapeutic benefit of carbon ion RT. Additionally, miRNA-654-3p in serum exosomes was considered a potential non-invasive biomarker to predict the efficacy of carbon ion RT in PCa (50). Likewise, Malla et al. collected 25 serum-derived extracellular vesicles-miRNAs from patients treated with RT. Five miRNAs were identified (let-7a-5p, miRNA-141-3p, miRNA-145-5p, miRNA-21-5p, and miRNA-99b-5p), but only let-7a-5p and miRNA-21-5p were overexpressed in high-risk PCa patients after RT.

More recently, miRNA-541-3p was studied in 33 PCa tissues and normal adjacent tissues before and after RT treatments. Interestingly, He et al. found that miRNA-541-3p enhances the radiosensitivity of PCa by inhibiting HSP27 expression and downregulating β-catenin (51).

However, further studies need to be carried out to confirm the miRNAs potential as new outcome biomarkers for PCa patients, as well as to validate the results already obtained, namely through larger patients’cohorts, due to the small sample size of studies-derived data available to date.

#### Effect of miRNA Expression on Radiation Response in PCa

The Radiation Therapy Oncology Group (RTOG) has performed several potential trials in the field of RT, exploring tissue-based molecular biomarkers with predictive or prognostic value.

More recently, Croce et al. revealed data on the miRNAs potential in cancer, and then other studies demonstrated that the ectopic modulation of specific miRNAs can influence the cancer hallmarks by deregulating its mechanisms (52, 53). In comparison with invasive methods, miRNAs, whose origin seems to be specific from tissue, are very stable and directly detectable in circulating biofluids (54). Also, miRNAs can be isolated and purified from serum, plasma, urine, saliva, peripheral blood cells, among other biological samples (55). Also, miRNAs can circulate in the interstitial fluids and bloodstream through membrane-bound vesicles, such as exosomes (50–90 nm) and microvesicles (1 μm), and even in non-vesicles, such as the ribonucleoprotein complex, which corresponds to the main mechanism. Indeed, accumulating evidence identified circulating miRNAs in apoptotic bodies, exosomes, high-density lipoprotein, and RNA binding proteins as a form of a cell-to-cell communication channel (56).

Circulating miRNA are evolutionarily conserved across species and can be measured easily and efficiently using real time quantitative protein chain reaction (RT-qPCR), microarray platforms, nanostring techniques, next-generation sequencing (NGS) and biosensors (56). Furthermore, evidence reveals that tumor-associated signature of miRNAs allows to discriminate different cancer subtypes and pathologies by using high-quality measurement techniques. Such finding can significantly contribute to the selection of a more efficient therapeutic approach (57, 58). Indeed, since miRNAs are involved in different cancer mechanisms, they can also be used in targeted therapy, however it continuous to be a challenge regarding stability of miRNAs and its tissue specificity and permeability (55). With the technological advance, such as in the field of nanotechnology, and with the raise in miRNA research, it is expected that, in the future, one of the therapeutic approaches for cancer may be the administration of synthetic anti-sense or mimics oligonucleotides (59, 60).

Several studies have shown the clinical usefulness of some miRNAs and their potential in therapeutic efficacy of RT (54, 61–63). Some miRNAs exhibit predictive value regarding the treatment response through sample analysis extracted from non-invasive liquid biopsies. Thus, miRNAs can give relevant data to achieve a proper patient therapeutic monitoring, as they promote an early detection of PCa relapse/progression, ultimately providing a better control of cancer (44). To underlined that this ability to predict whether a patient is responsive or nonresponsive to a particular treatment modality will allow the expansion of personalized medicine, with individual and personalized treatments being selected for a particular patient, avoiding the risks of toxicity, side effects and relapses. Moreover, “real-time” monitoring of miRNAs may provide an early identification of patients who are failing to radiation therapy response, offering the opportunity to try a more efficient alternative treatment (64). In 2008, it was published the first evidence of a miRNA signature that changed the response to RT (65). Subsequently, increasing evidence has been generated with the intent of discovering an “universal” miRNA molecular profile.

#### Function and Targets of miRNAs Involved in Radiation Response in PCa

The interaction of ionizing radiation with cells induces some biological responses, including direct DNA damage from ionization or indirectly by ROS generation. Then, different pathways are activated in an intent to repair the damaged DNA, induce cell cycle arrest or even cell death (66). As stated above, RT induce damages, including single-strand breaks (SSB) and DSB. These breaks can be restored by DNA repair pathways, such as base excision repair (BER), nucleotide excision repair (NER), mismatch Repair (MMR), nonhomologous end-joining (NHEJ) or homologous recombination (HR) (67). But radiation can also change the miRNA expression and consequently alters the levels of associated proteins. Most of these studies have been done *in vitro* using PCa cell lines, such as, PC3, DU145, LNCaP and 22Rv1.

**Figure 6:**
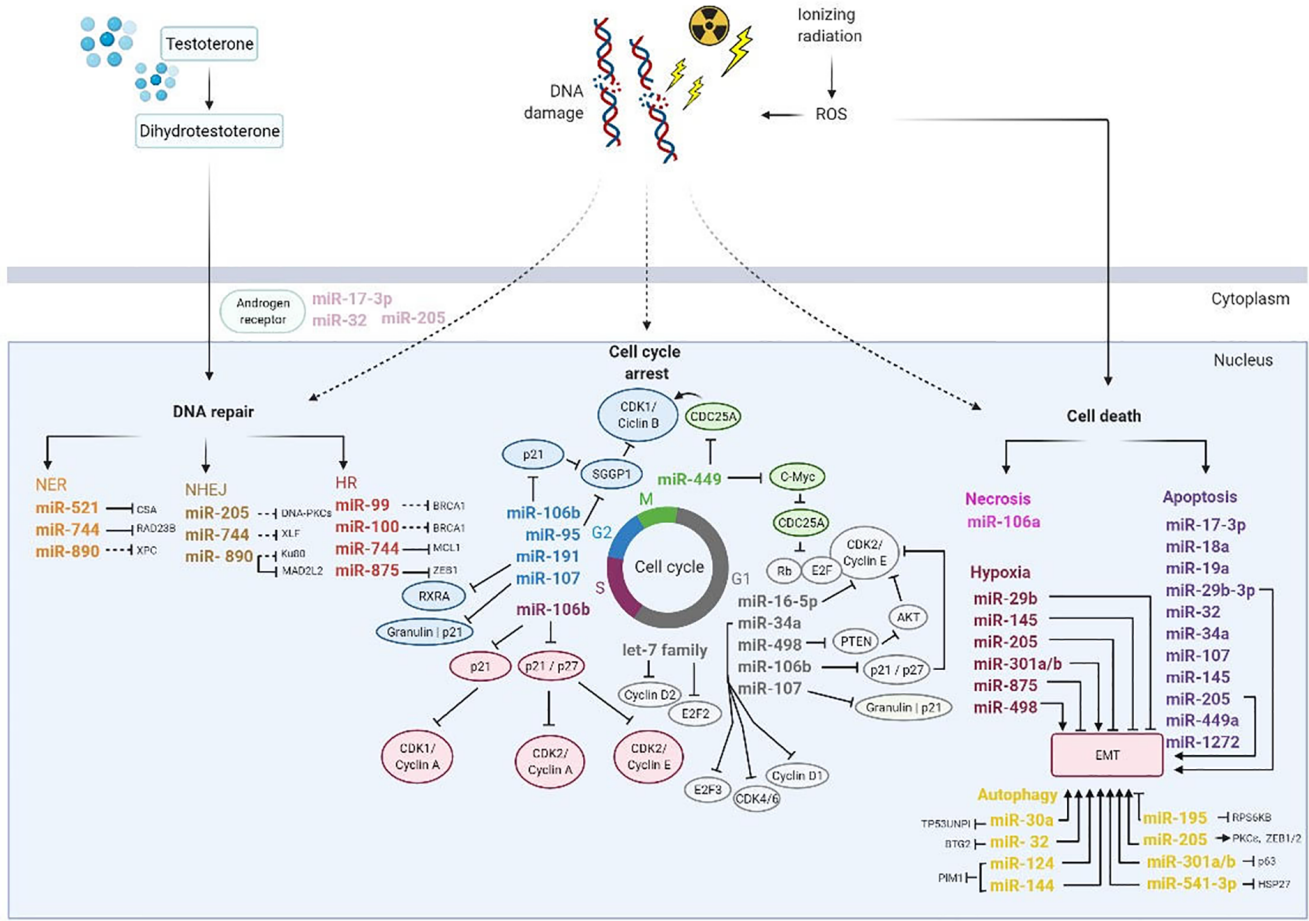
Overview of miRNAs involved on DNA damage repair, cell cycle arrest, and cell death induced by ionizing radiation. Thus, photon beams cause DNA damage directly or indirectly by reactive oxygen species (ROS). In order to repair DNA damage, the cell activate DNA damage response pathways including nucleotide excision repair (NER), non-homologous end joining (NHEJ) and homologous recombination (HR). Additionally, miRNAs regulated cell cycle progression to allow DNA damage repair and depends on cyclin dependent kinases (CDKs), cyclins and transcription factors family EF2. Also, miRNAs are influenced by several factors in the tumor microenvironment such as hypoxia and epithelial mesenchymal transition (EMT) and play an important role in biological processes as apoptosis and autophagy. Consequently, hypoxia promotes DNA repair by transcription of the androgen receptor expression. AKT, Protein kinase B; CDC25A, Cell division cycle 25 A; G1 and G2, transition phases of the cell cycle; HSP27, Heat shock protein 27; M, Mitosis; PTEN, Phosphatase and TENsin homolog; Rb, Retinoblastoma protein; RXRA, Retinoid X receptor alpha; S, phase S; SGGP1, Sphingosine-1-phosphate phosphatase 1. Black inhibition line displays direct targeting; black dashed-inhibition line displays indirect targeting; arrow display an induction of tumor microenvironment.

MiRNA are involved in the management of such different cell processes (**Figure 6**). For example, MiRNA-99a, a member of miRNA-99 family, and miRNA-100 have a role in DNA repair. The inhibition of this miRNAs will prevent p53 dependent apoptosis, increasing the recruitment of DNA repair proteins (BRCA1, RAD51), consequently influencing SWI/SNF-related matrix-associated actin-dependent regulator of chromatin subfamily A member 5 (SMARCA5) and spinal muscular atrophy with respiratory distress type 1 (SMARD1) in LNCaP, PC3 and DU145 cells after irradiation exposure (68, 69).

Josson et al. reported that miRNA-521 modulates the radio sensitivity of LNCaP cells by specifically restoring DNA repair protein, Cockayne syndrome protein A (CSA) and manganese superoxide dismutase (MnSOD), an anti-apoptotic enzyme. If miRNA-521 is overexpressed it will further sensitize cells to RT contributing to a raise in RT efficacy (65).

Furthermore, miRNA-890 is downregulated in LNCaP, PC3 and DU145 cells and targets mitotic pathways composed of several regulators, including mitotic arrested deficient 2 like 2 (MAD2L2), WEE1 kinase, xeroderma pigmentosum complementation group C (XPC), and KU80 proteins. Also, Hatano et al. revealed that miRNA-744-3p can directly influence RAD23 Homolog B, Nucleotide Excision Repair Protein (RAD23B) in LNCaP, PC3 and DU145 cells. Both miRNAs are involved in DDR systems induced by irradiation, such as DNA DSB repair and NER pathways (70, 71). El Bezawy et al. mentioned that miRNA-875-3p inhibits HR pathway in PC3 and DU145 cells by controlling checkpoint kinase 1 (CHK1) expression and zinc finger E-box-binding homeobox (ZEB), which have impact on EMT (72).

Similarly, there are some miRNAs involved in cell cycle arrest. MicroRNA–16–5p is located at chromosome 13q14 and it is downregulated in LNCaP cells. Wang et al. showed that this miRNA is a tumor suppressor and is involved in PCa onset. The overexpression of miRNA-16-5p is linked to cell proliferation suppression and modulates the Cyclin D1/E1-pRb-E2F1 pathway, inducing G0/G1 phase arrest after irradiation, which consequently increase the radio sensitivity in LNCaP cells (34).

In G2/M phase, elevated levels of miRNA-95 promote radio resistance in PC3 cells. The target of this miRNA is associated with SGPP1, an antagonist of sphingosine-1-phosphate signaling (S1P) that is responsible to protect against ionizing radiation-induced cell death. Briefly, SGPP1 suppresses the G2/M checkpoint, while increases proliferation, invasiveness, and the migratory capabilities of cancer cells (47, 73).

Also, miRNA-106 has been implicated in several pathways involved in raising the PCa cells radioresistance. Hoey et al. analyzed miRNA-106a and concluded that it is overexpressed in PC3 and DU145 cells and is significantly increased in high-grade than low-to-intermediate-grade cancer. The miRNA-106a targets lipopolysaccharide-induced TNF-α factor (LITAF), which is responsible to confer a radioresistant phenotype that increases cell survival and proliferation after irradiation (74). Li et al. showed that miRNA-106b have a novel role in RT due to its involvement in p21-activated cell cycle arrest regulation. Therefore, an inhibitory approach with addition of anti-miRNA-106b may reduce the miRNA-106b levels and will then change the p21 levels. After irradiation, a marked decreased in miRNA-106b expression was also stated in LNCaP cells (48, 73, 75), along with a correlation between miRNA-106b and Caspase-7 (76, 77).

Mao et al. showed that miRNA-449a targets c-Myc in LNCaP, PC3 and DU145 cells, which controls cdc2/Cyclin B1 cell cycle signal. This miRNA also enhances radiation-induced growth inhibition, radiation-induced G2/M arrest, and apoptosis by modulating the Cdc25A/Rb/E2F1 pathway. Likewise, c-Myc, which controls Cdc25A expression, is a miRNA449a target and is involved in PCa progression and its expression decreases after cells are submitted to radiation. So, when miRNA-449a is overexpressed, it promotes radio sensitivity *in vitro* by triggering destabilization and decreasing the expression of c-Myc and increasing both G2/M arrest and apoptosis (78, 79).

Moreover, miRNA-191 was correlated with radiation response *in vitro* and *in vivo.* Normally, this miRNA is overexpressed in PCa and it was related with radiation resistance through interaction with a novel target, retinoid X receptor alpha (RXRA) in PCa cell lines (PC3 and DU45). Low levels of RXRA expression was linked with a higher risk of distant relapse following RT. Mechanistically, miR-191 also effects cell cycle distribution and proliferation, reducing G2-M phase arrest post-radiation (80).

More recently, miRNA-107 has been related with radiation response of PCa. Lo et al. found that miRNA-107 regulated granulin and is downregulated in response to ionizing radiation in PC3 cells. MiRNA-107 was downregulated in PCa cells and tissues in comparison with normal prostate cells, but when is overexpressed, blocked granulin and promoted the radiosensitivity in PC3 cells. Mechanistically, miR-107 induced G1/S phase arrest and G2/M phase transit. Besides, also enhancing delayed apoptosis through suppression of p21 and CHK2-phosphorylation (81).

According to Duan et al., miR-498 is linked to PCa cells proliferation, radio sensitivity, invasion, and migration. After exposure to ionizing radiation, this miRNA is under expressed and induces radiation resistance in LNCaP and DU145 cells by reducing radiation-induced apoptosis through BAX and Bcl-2 expression regulation (82). Additionally, miR-498 is related to an important cell cycle regulator, phosphatase and TENsin homolog (PTEN), that suppresses the protein kinase B (AKT) signaling pathway, inhibits cell cycle progression, and affects ionizing radiation-induced apoptosis triggered by caspase 3/7 activity. In addition, with PTEN and AKT inhibition, EMT changes through influence of a raised expression of vimentin and a decreased of E-cadherin (82). Also, delays in response to DNA damage trigger cell death through several mechanisms, such as apoptosis, senescence and autophagy (83). Hsu et al. suggested that miR-18a acts as an oncomiRNA in cancer progression and it is upregulated in 22Rv1, PC3, LNCaP and DU145 cells. MiR-18a is related to STK4, a pro-apoptotic kinase that mediated AKT apoptosis cascade by phosphorylate Caspase 9 and Bad (84). In addition, Yang et al. showed that miR-18a were modulated by growth arrest-specific 5 (GAS5), which protects from radiation and promotes apoptosis when low expressed (85).

Recently, miR-541-3p has been investigated in radiation response in PCa tissue samples and cell lines. MiR-541-3p has low expression in PCa tissues, however, when submitted to RT is overexpressed in PCa cells (LNCaP, DU-145, PC3, and PrEC). Thus, using the mimic approach, miRNA-541-3p interacted directly with HSP27 and increased the radiosensitivity by enhanced apoptosis (51).

In a loss-of-function setting, miR-541-3p knockdown increased the proliferative potential and decreased the apoptotic rate of irradiated cells, ultimately reducing cell radiosensitivity. Conversely, miR-541-3p overexpression by miRNA mimic increased cell sensitivity as a result of a reduction in cell viability and colony formation, paralleled by increased apoptosis. Mechanistically, HSP27, validated as a direct target of the miRNA, was proposed as the potential mediator of miR-541-3p-induced radiosensitization, as suggested by rescue experiments showing a partial reversion of miRNA biological effects upon HSP27 ectopic overexpression.

Also, miR-29b expression or deletion was observed in tumor tissues and cell lines. Mao et al. demonstrated that miR-29b-3p improves radiation-induced cell apoptosis and sensitizes LNCaP cells to radiation by targeting Wnt1-inducible-signaling protein 1 (WISP1). Also, this miRNA was found to be a regulator of EMT and inhibits the PCa cells proliferation and invasion by controlling different targets, among them MCL-1, MMP-2, DNMT3B, and AKT3 (86).

MiRNA-19a was also analyzed in LNCaP, PC3 and DU145 cell lines and it was found downregulated in p53 positive radiosensitive LNCaP cells. Thus, it was suggested that miRNA-19a inhibition can provide a new therapeutic strategy for radioresistant PCa with mutated p53. This miRNA is related with prostate transmembrane protein, androgen-induced l(*PMEPA1*), and tumor protein p53 inducible nuclear protein 1 (*TP53INP1*) (87). Another miRNA, miRNA-17-3p was found at reduced amounts in PC3 cells and there have been some suggestions that this miRNA promotes carcinogenesis, by inhibition of mitochondrial antioxidant enzymes, such as manganese superoxide dismutase (MnSOD), glutathione, peroxidase 2 (Gpx2), and thioredoxin reductase 2 (Trx2) (88). In this context, Xu et al. provided a proof-of-concept evidence that miR-17-3p upregulation influences the radiotherapeutic efficiency through suppressing ionizing irradiation-mediated antioxidant responses, and in turn contributing to a raise in ROS level (89).

MiRNA-32 regulates DAB2 interacting protein (DAB2IP) and may contribute to the radioresistant PCa cells due to reduced ionizing radiation-induced cell apoptosis. Moreover, when this miRNA is overexpressed in LNCaP, PC3 and DU145 cells, it inhibits the expression of Bim protein, a pro-apoptotic member of the BCL-2 family and induces autophagy by targeting DAB2IP (48, 90). MiRNA-32 is also regulated by androgen and it has been implicated to another target gene, B-cell translocation gene 2 (BTG2), which is associated with PCa aggressiveness (91). Another functional study in DU145 and PC3 cell lines showed that miRNA‐124 or miRNA‐144 overexpression inhibit hypoxia‐induced autophagy and enhance radiosensitivity by regulating PIM1 (92).

A recent study on miR-1272 has revealed a relation with radio sensitivity of DU145 cells due to a consistent reduction of clonogenic cell survival mediated by miR-1272 upon irradiation. Authors transformed cells in a manner of gain-of-function using miR-1272 mimics. They found that besides reduced tumor growth and enhanced response to RT, miR-1272 affected the GFR/AKT/ERK1 pathways, ultimately affecting migration, invasiveness, and preventing EMT, all essential steps of the metastatic cascade (49).

Several studies have also shown that miRNA-145 overexpression sensitizes LNCaP and PC3 cells to ionizing radiation. This miRNA suppresses DNA (cytosine-5-)-methyltransferase 3 beta (DNMT3b), which have a crucial role in carcinogenesis, influencing PCa cells cycle, apoptosis, growth, and migration. Some data suggest that the overexpression of miRNA-145 can improve radio sensitivity through DNA DSB downregulation and directly targeting oncogenes (46, 65, 93). More recently, El Bezawy et al. described that miRNA-145 mimics can silence and deregulate the Speckle-type pox virus and zinc finger protein (POZ) protein (SPOP), causing an increase in PCa cells radio sensitivity by decreasing RAD51 and CHK1 expression and targeting ZEB1, that increases E-cadherin expression (94).

Another important feature linked to cancer cells is hypoxia, responsible for promoting tumor progression and the aggressive phenotype (95). In this sense, miRNA-301a and 301b, members of miRNA-301 family, also present clinical interest. It is known that miRNA-301a is an oncomir and it has been proposed that miRNA-301b can act as a tumor suppressor in LNCaP, PC3 and DU145 cells. However, in study of Wang et al. both these miRNAs were related to hypoxia and led to a decrease in autophagy in LNCaP, PC3 and DU145 cells by targeting N-myc downstream-regulated gene 2 (NDRG2). If these miRNAs are overexpressed, they may induce radio resistance in PCa cells by decreasing NDRG2 that suppresses EMT (96–98). Also, cancer change EMT. Several pathways have been clarified as involved in EMT deregulation, namely those linked to a control in transcription factors and epithelial specific markers, such as a decrease in cytokeratins and E-cadherin, and an increase in mesenchymal markers, such as fibronectin, N-cadherin, and vimentin (99). Indeed, El Bezawy et al. demonstrated that miRNA-875-5p is under expressed in PC3 and DU145 cells. MiRNA-875-5p is directly related to E-cadherin, with neutralization of EMT and improvement of radiation response through targeting epidermal growth factor receptor (EGFR), being these some of the major roles of E-cadherin. Also, it is involved in HR to repair DNA by regulating checkpoint kinase 1 (CHK1) expression and ZEB 1 (72, 100).

Also, MiR-34a and let-7 family (let-7a, let-7b, let-7c, let-7d, let-7e, let-7f, let-7g and let-7i) appeared upregulated following fractionated irradiation in LNCaP and PC3 cells, but not in DU145 cells. All these miRNAs are related to p53 gene, but only miRNA-34a has been proposed to be used as a radio sensitivity predictor, as it targets cyclin E2, besides to also interact with EMT (87, 101). Other studies suggest that miRNA-34 can be used to potentiate the therapeutic effect, as it is overexpressed in LNCaP and underexpressed in PC3 cell line (65, 102, 103). Also, other study should that let-7 family expression was downregulated in LNCaP, PC3 and DU145 cells and revealed to be able to regulate the expression of RAS oncogene, such KRAS and c-Myc (104). Furthermore, Dong et al. demonstrated that let-7a induced cell cycle arrest at the G1/S phase modulating the expression of E2F Transcription Factor 2 (E2F2) and G1/S-specific cyclin-D2 (CCND2) (105).

MiRNA-205 is under expressed and mediates autophagy, which is an important mechanism that can influence the LNCaP, PC3, DU145 cells radio sensitivity (106). Autophagy acts like a protective mechanism of PCa cells to stressful conditions, including radiation-induced cell apoptosis (107). Also, a potential direct functional target of miRNA-205 is tumor protein p53-inducible nuclear protein 1 (TP53INP1), which can interact with other protein families, such as Light chain 3 (LC3) and autophagy-related protein 8 (ATG8), thereby promoting autophagy and apoptosis targeting several cells signaling components, namely mitogen-activated protein kinase (MAPK) and androgen receptor (106, 107). Furthermore, miRNA-205 is also important to support the basal membrane in prostate epithelium, protein kinase C epsilon (PKCϵ) and ZEB1 expression, proteins involved in EMT (40). In the same context, miRNA-30a has been able to suppress autophagy and enhance radiosensitivity of PCa cells by targeting TP53INP1 (107).

Still related to autophagy, miRNA-195 is linked to PC3 and DU145 progression by targeting ribosomal protein S6 kinase B1(RPS6KB1), with its overexpression being responsible to enhance the RT efficacy through T cell by blocking the PD-L1 immune checkpoint, which is related to regulation of cytokines secretions in the tumor (108).

McDermott et al. showed that miR-4284 negatively regulates ring finger protein, LIM domain interacting (RLIM) and RasGEF domain family member 1A (RASGEF1A) genes. These genes are associated with RT resistance and oncogenesis. Authors also underlined that miR-4284 is down-regulated in RR-22Rv1 and AMC-22Rv1 cells, and stated a non-significant trend towards the acquisition of age-related radio resistance. Besides, another five miRNAs (miR-210, miR-23a, miR23b, miR-24, and miR-29) were identified in both hypoxic and isogenic radioresistant 22Rv1 models, when compared to the more radiosensitive WT-22Rv1 cell line (109).

Thus, a set of evidence in PCa treatment show that RT can significantly change the miRNA expression levels, but only a few studies investigate the impact of miRNA expression on radiation response in PCa (**Table 1**).

**Table 1:** MiRNA expression in radiation response in prostate cancer cell lines.

miRNA	Cell line used	Function	miRNA expression before irradiation	Functional role	Therapeutic strategy	D	P	References
hsa-miRNA-16-5p	LNCaP	TS	↓	RR	Mimicking	✓	✓	(34)
hsa-miRNA-17-3p	PC3	–	↓	RR	Mimicking	✓	–	(89)
hsa-miRNA-18a	22Rv1, PC3, LNCaP, DU145	OM	↑	RR	Antagomirs	–	✓	(84, 85)
hsa-miRNA-19a	LNCaP, PC3, DU145	OM	↑	RR	Antagomirs	✓	✓	(87)
has-miRNA-29b-3p	LNCaP	TS	↓	RR	Mimicking	✓	✓	(86)
Has-miRNA-30a	LNCaP, DU145	TS	↓	RR	Mimicking	✓	✓	(107)
hsa-miRNA-32	LNCaP, PC3, DU145	OM	↑	RR	Antagomirs	✓	–	(48, 90)
hsa-miRNA-34a	LNCaP, PC3, DU145	TS	↓	RR	Mimicking	✓	✓	(87)
hsa-miRNA-95	PC3	–	↑	RR	Antagomirs	✓	✓	(47, 73)
hsa-miRNA-99a	LNCaP, PC3, DU145	TS	↓	RR	Mimicking	✓	✓	(68)
hsa-miRNA-100	LNCaP, PC3, DU145	TS	↓	RR	Mimicking	✓	✓	(68)
hsa-miRNA-106a	PC3, DU145	OM	↑	RR	Antagomirs	✓	✓	(74, 110)
hsa-miRNA-106b	LNCaP	OM	↑	RR	Antagomirs	✓	–	(48, 75)
hsa-miRNA-107	PC3	–	↓	RR	Mimicking	✓	✓	(81)
has-miRNA-124	PC3, DU145	TS	↓	RR	Mimicking	✓	✓	(92)
Has-miRNA-144	PC3, DU145	TS	↓	RR	Mimicking	✓	✓	(92)
hsa-miRNA-145	LNCaP, PC3	TS	↓	RR	Mimicking	✓	✓	(46, 65, 70, 93, 94)
Has-miRNA-191	PC3, DU145	OM	↑	RR	Antagomirs	–	✓	(80)
hsa-miRNA-195	PC3, DU145	TS	↓	RS	Mimicking	✓	✓	(108)
hsa-miRNA-205	LNCaP, PC3, DU145	TS	↓	RR	Mimicking	✓	✓	(40, 106, 107, 110)
hsa-miRNA-301a	LNCaP, PC3, DU145	–	↑	RR	Antagomirs	–	✓	(97, 110)
hsa-miRNA-301b	LNCaP, PC3, DU145	–	↑	RR	Antagomirs	–	–	(97)
hsa-miRNA-449a	LNCaP, PC3, DU145	TS	↓	RR	Mimicking	✓	–	(78)
hsa-miRNA-498	LNCaP, DU145	–	↑	RR	Antagomirs	–	✓	(82)
hsa-miRNA-521	LNCaP	–	↓	RR	Mimicking	–	–	(65)
hsa-miRNA-541-3p	LNCaP, DU-145, PC3, and PrEC	TS	↓	RR	Mimicking	✓	✓	(51)
hsa-miRNA-744-3p	LNCaP, PC3, DU145	OM	↑	RR	Antagomirs	–	–	(70, 71)
hsa-miRNA-875-5p	PC3, DU145	TS	↓	RR	Mimicking	–	–	(72)
hsa-miRNA-890	LNCaP, PC3, DU145	–	↓	RR	Mimicking	–	–	(70)
hsa-miRNA-1272	DU145	TS	↓	RR	Mimicking	–	✓	(49)
hsa-miRNA-4284	22Rv1	–	↓	RR	Mimicking	–	–	(109)
Let-7 family	LNCaP, PC3, DU145	TS	↓	RR	Mimicking	–	✓	(87, 104)

D, Diagnostics; P, Prognostics; TS, Tumour suppressor miRNA; OM, Oncogenic miRNAs; RR, Radioresistant; RS, Radiosensitive; ✓, present; –, absent of information; ↑, increased expression; ↓, decreased expression.

Anyway, and despite the accumulating evidence on this subject, it is important to mention that miRNA expression levels can be modified following PCa irradiation (29, 87, 111, 112). But, despite such alterations in miRNA expression patterns are inconsistent, even within the same cell line, because it largely depends on radiation dose and recovery time post-irradiation of cells (15), it is also a matter of high focus nowadays.

## Conclusion and Future Perspectives

Despite the relative few numbers of studies exploiting the miRNAs relation with radiation response of PCa cells, this subject has been progressively explored and it continuous to be a challenge regarding the role of miRNAs as predictive markers for therapeutic targets.

One limitation of the miRNAs signature is linked to the inconsistency found among studies, mostly attributed to the methodologies applied: clinical trials/experimental studies, therapeutic conditions, pathology type, cell type, among others. Thus, to overcome this drawback, further studies must be designed to get high-quality, reproducible, and valid representative miRNAs to achieve results capable of promoting a patient-tailored treatment. Also worth of note is that most studies analyzed the potential role of miRNAs *in vitro*, so that new experiments should be done *in vivo* or in human tissue samples to support such findings. Thus, the selection of the most appropriate miRNAs remains a challenge.

In short, it has been shown that several miRNAs that modulated the cell response to ionizing radiation. Thus, miRNAs can be applied in therapy to reduce the radio resistance of cells through modulation of cell pathways and biological processes. However, larger and prospective studies are essential to define the value of miRNAs as therapeutic adjuvant to RT. Also, in this context, and since the number of studies is increasing, it is important to ensure a proper organization of data by creating databases of miRNA expressions for cancer research. In the future, we hope to find miRNA target relevant in daily clinical practice, with those capable of predicting the RT efficacy response being highly valuable in RT treatment management.

## Author Contributions

All authors have contributed equally to this work. All authors contributed to the article and approved the submitted version.

## Conflict of Interest

The authors declare that the research was conducted in the absence of any commercial or financial relationships that could be construed as a potential conflict of interest.

## Publisher’s Note

All claims expressed in this article are solely those of the authors and do not necessarily represent those of their affiliated organizations, or those of the publisher, the editors and the reviewers. Any product that may be evaluated in this article, or claim that may be made by its manufacturer, is not guaranteed or endorsed by the publisher.
